# Multiple paleofire proxy metrics from tropical lake sediment and soil in the Greater Serengeti Ecosystem

**DOI:** 10.1177/09596836251340882

**Published:** 2025-06-26

**Authors:** Colin J. Courtney Mustaphi, Sandra O. Camara-Brugger, Nathan J. Chellman, Samuel Muñoz, Rebecca W. Kariuki, Maja Damber, Anna Shoemaker, Anneli Ekblom, Linus Munishi, Paul Lane, Rob Marchant, Oliver Heiri

**Affiliations:** 1 Geoecology, Department of Environmental Sciences, University of Basel, Switzerland; 2 York Institute for Tropical Ecosystems, Department of Geography and Environment, University of York, UK; 3 Knowledge Core GmbH, Switzerland; 4 Division of Hydrologic Science, Desert Research Institute, USA; 5 Department of Marine & Environmental Sciences, Northeastern University, USA; 6 Department of Civil & Environmental Engineering, Northeastern University, USA; 7 School of Sustainability, College of Global Futures, Arizona State University, USA; 8 Terra Archaeology Limited, Canada; 9 Department of Archaeology and Ancient History, Uppsala University, Sweden; 10 School of Life Sciences and Bioengineering, The Nelson Mandela African Institution of Science and Technology, Tanzania; 11 Department of Archaeology, University of Cambridge, UK; 12 School of Geography, Archaeology and Environmental Studies, University of the Witwatersrand, South Africa

**Keywords:** black carbon, charcoal, combustion products, geoarchives, kopje, paleofire proxies, proxy comparison

## Abstract

Black carbon is a paleofire proxy that has been measured from glacial ice, snow, soils and lake sediments, though relatively few comparisons have been made with other fire indicators in sedimentary geoarchives. Microscopic charcoal, quantified from palynological microscope slides and macroscopic charcoal, quantified from wet-sieved deposits, are the most commonly applied methods for paleofire interpretation of Quaternary sediments. This research explores the down-profile patterns across three paleofire proxies (refractory black carbon, microscopic and macroscopic charcoal) and potential paleofire interpretations from a sediment core dating to the last centuries from Speke Gulf, Lake Victoria, and a young soil profile from a kopje located in the surrounding watershed in Serengeti National Park, Tanzania. The results of three paleofire metrics show similar trends within each site, with a positive trend across all metrics and increasing variability with increased measurement values (heteroscedastic). Notably, refractory black carbon (rBC) concentrations are two orders of magnitude higher in lake sediment samples compared to soil samples. rBC is positively correlated with both microscopic and macroscopic charcoal values and the overall profile patterns down the sediment core are similar, with the exception of the rBC increases from 2.5 to 0 cm depth that may result from increased fossil fuel combustion. The Speke Gulf rBC measurements are in an intermediate range between those published from glacial ice and other lake sediments. New rBC records from different ecosystems and temporal scales will provide paleofire insights and potential to interpret source areas and depositional patterns. The exploration of soil archives offers the potential to exploit semi-arid ecosystems and archaeological sites that have no nearby traditional paleoenvironmental study site targets.

## Introduction

Black carbon (BC) is an aerosol emitted from the burning of both biomass and fossil fuels (Bond et al., 2013; Brugger et al., 2023), and is transported in the atmosphere and then deposited at local-to-hemispheric distances from the emission sources (Coppola et al., 2022; Kuhlbusch, 1998). Quantification of past emissions informs global climate model parameterization of black carbon forcing (Eckhardt et al., 2023; Li et al., 2024) and paleoenvironmental records of black carbon deposition are a useful proxy measurement for emissions prior to 20th-century observations, especially from the Global South where fewer instrumental and paleoenvironmental records are available (Liu et al., 2021; Penner et al., 1993; Wang et al., 2014). Several measurement techniques for black carbon in paleoenvironmental geoarchives are available alongside other proxies used for paleofire reconstructions, such as concentrations of microscopic and macroscopic charcoal (Conedera et al., 2009; Rose and Ruppel, 2015). Few intercomparisons of multiple fire proxies are published in tropical environments (Arienzo et al., 2019; Masiello, 2004; Thevenon et al., 2010), these, nonetheless, can contribute to our understanding of past human-environment modifications to landscapes and socio-ecological processes (Dabengwa et al., 2022; Gillson et al., 2019, 2025; Sayedi et al., 2024).

Different measurement techniques are sensitive to distinct regions of the BC continuum ranging from char to highly condensed soot (Hammes et al., 2007; Hedges et al., 2000; Jones et al., 1997; Petzold et al., 2013). This study focuses on refractory black carbon (rBC), which represents the most condensed, sub-micron sized BC particles emitted during combustion that are typically measured using laser-induced incandescence methods (Chellman et al., 2018). Analyses of rBC from geoarchives have been undertaken from glaciers in polar and high elevation regions (Arienzo et al., 2017; Goto-Azuma et al., 2025; McConnell et al., 2007, 2021) with fewer records from snow (Cordero et al., 2022; Osmont et al., 2018) or lake sediments (Arienzo et al., 2019; Brugger et al., 2024; Camara-Brugger et al., 2025; Chellman et al., 2018). While BC determination is heavily dependent on the specific methodology for its quantification, measurements of high-refractory soot BC produced in the gas phase of high temperature combustion (i.e. relatively similar to rBC) have been made from sediments and soils, for example, in China (Cong et al., 2013; Han et al., 2010, 2015), Europe and the Arctic (Bogdal et al., 2011; Elmquist et al., 2007, 2008) and North America (Louchouarn et al., 2007) and elemental carbon records have been published for Africa (Mekonnen et al., 2022). Most sediment BC records to date have been developed using thermal/optical transmittance (TOT) or chemothermal oxidation (CTO) techniques used to determine the total elemental carbon (EC) concentration – comprised of both the char and soot portions – of a sample. Previous work has shown that Single-Particle Soot Photometer (SP2) measurements of rBC are comparable with the soot component of TOT/CTO methodologies as expected given the SP2 is most sensitive to refractory soot-like BC particles (Chellman et al., 2018). Additional analyses from other geoarchives, such as soil (Agarwal and Bucheli, 2011; Cornelissen et al., 2005), have the potential to complete geographical gaps, notably in tropical regions and areas that were modified by early anthropogenic fire uses and will enhance the use of BC as a paleofire proxy to understand flammable ecosystems, such as tropical savannahs (Bowman et al., 2020; Courtney-Mustaphi et al., 2018; Shetler, 2007).

Several paleofire metrics have been developed for the analysis of depositional environments and interpretation of past fires and microscopic and macroscopic charcoal are the most frequently used methods for lake sediments (Harrison et al., 2022; Hawthorne et al., 2018). Advancements in charcoal morphometrics (Vachula et al., 2024), size fraction (Temoltzin-Loranca et al., 2023), morphological analyses (Courtney Mustaphi et al., 2025a; Feurdean et al., 2023), anthracology (Dussol et al., 2017; Kabukcu and Chabal, 2021), spectroscopy techniques (Maezumi et al., 2021; Minatre et al., 2024) and (bio)geochemical analyses (Battistel et al., 2017, 2018; Karp et al., 2023) have increased the potential to investigate more ecological and socio-ecological aspects of paleofire records. Quantification of multiple paleofire proxies from a single site offers an opportunity for intercomparison (Courtney Mustaphi and Pisaric, 2014, 2018; Isaksson et al., 2003). Comparison of several paleofire proxies subsampled from contiguous co-located subsamples is an opportunity to assess the congruence of proxy values along the range of fire-product particle sizes, and to resolve differences between the indicators and interpretation of the fire signals of the records (Brugger et al., 2022; Han et al., 2011; Ruppel et al., 2015). The aim of this study is to measure the range of rBC values in sediments from a large lake and a soil site within the watershed in equatorial eastern Africa to compare and interpret rBC with two established charcoal-based techniques from the same samples. Because of the different particle sizes of the fire products, we expect the paleofire signals of rBC, microscopic charcoal and macroscopic charcoal in sediments to reflect different source areas of different spatial scales. Different source areas, entrainment and transport mechanisms, depositional catchments and taphonomic factors each influence paleofire indicators (Courtney Mustaphi et al., 2022; Peters and Higuera, 2007; Vachula and Rehn, 2023), in the absence of major industrial sources or rBC contribution from fossil fuel burning. In general, the quantity of combustion products and amount deposited in a depositional environment depends on several factors (Bodí et al., 2014; Scott, 2010). Larger combustion products tend to be transported shorter distances (Peters and Higuera, 2007; Vachula and Rehn, 2023); convection, advection and runoff rates vary during and post-fire; entrainment and transport are influenced by charcoal morphologies (Pisaric, 2002); and the syndepositional processes of the combustion products vary across depositional environments (Whitlock and Millspaugh, 1996). In addition, significant contributions from industrial fossil fuel burning would affect rBC paleofire signals but not microscopic and macroscopic charcoal. We therefore expect the different paleofire indicators in our study to show similar longer-term trends, representing past changes in regional to local fire signals around the sites; some variation related to the combustion product particle size and transport distances; and potential differences due to varying contributions from hydrocarbon combustion to the rBC values.

## Material and methods

**Figure 1.**
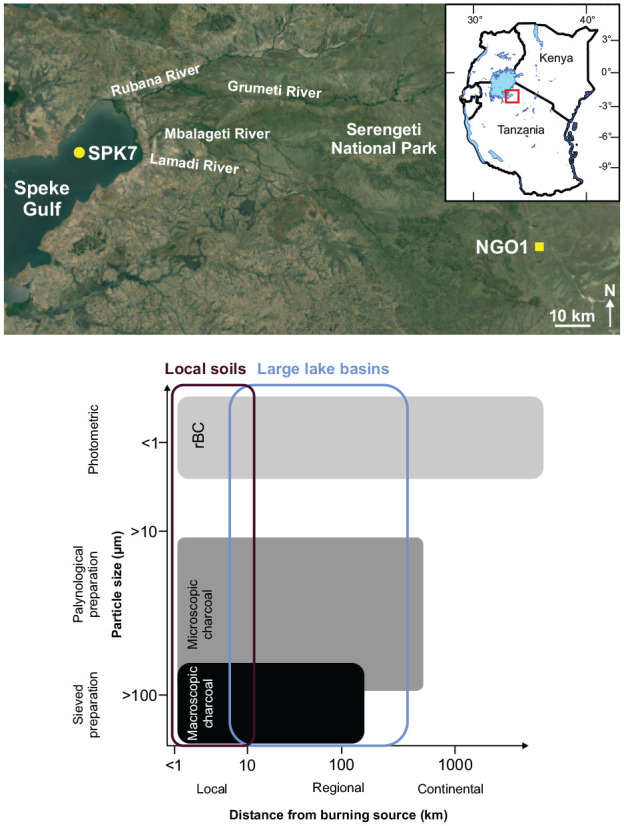
Inset map of eastern Africa and study area (red square) with sample collection locations (sediment core, yellow circle; soil pit, yellow square) and main river catchments that flow to Speke Gulf, Lake Victoria. Heuristic diagram summary of the nested but different theoretical spatial scales (horizontal axis) of paleofire proxy indicators (greyscale boxes) and associated pyrogenic particle sizes and measurement techniques (vertical axis; Conedera et al., 2009; adapted from Brugger et al., 2022; Kehrwald et al., 2016). Basemap: Google Earth, 2023. http://earth.google.com/web/. *Note*. Please refer to the online version of the article to view this figure in color.

Lake sediment samples from a gravity core from Lake Victoria (SPK7, *n* = 43) and subsurface samples from a soil pit in a Serengeti kopje (NGO1, *n* = 15) were used in this study. The soil sample was collected from within the watershed of Speke Gulf, approximately 140 km distant from the coring site (Figure 1). Soil samples were analysed to determine the viability of soils as a geoarchive of rBC and to compare with charcoal paleofire indicators. Sample collection sites are within the Speke Gulf drainage catchment that is predominantly covered with grassy and woody savannahs and rural agriculture. Currently, fires mainly occur in the protected areas during the bimodal drier seasons and easterly winds are common transporting smoke.

### Study sites

Speke Gulf (1135 m asl) is a large, relatively flat and shallow (~10 m), embayment of Lake Victoria, Tanzania, that is ~15–20 km wide (north-south) and 100 km long (east-west; Figure 1; Bootsma et al., 2003; Scholz et al., 1990). The river watersheds of eastern Speke Gulf (~740 km^2^) consist of rural agriculture and small urban settlements, shrublands and woody or grassy savannahs (Awange and Ong’ang’a, 2006; Mugo et al., 2020). The primary inflows to eastern Speke Gulf include the Rubana-Grumeti and Mbalageti Rivers and several smaller rivers that flow through a gently tilted watershed from up to ~1800 m asl through central Serengeti and the Western Corridor (Figure 1; Scoon, 2018; Sinclair et al., 2008). Most of the savannah in the protected areas near Speke Gulf currently burn at average return intervals of 1–4 years (Eby et al., 2015). Variability of fire is high, from twice per year in the most productive grasslands to >15 years between fires in mesic woody savannahs (Dempewolf et al., 2007; Hempson et al., 2018; Probert et al., 2019). Although ignitions may be more frequent outside of protected areas, fires are more limited in extent due to fragmentation of fuel connectivity and differential fire management strategies (Beale et al., 2018; Butz, 2009; Kamau and Medley, 2014). In the Speke Gulf catchment, the vegetation that fuels fire is predominantly savannah graminoids (Temoltzin-Loranca et al., 2023) and a smaller contribution from woody shrubs and trees (Battistel et al., 2017; Courtney Mustaphi et al., 2022; Smith and Hudak, 2005).

Kopjes and inselbergs support distinct habitats that are intermittent within the savannahs and shrublands and cover <1% of the total area of Serengeti National Park (Byrom et al., 2015a, 2015b; Scoon, 2018; Timbuka and Kabigumila, 2006). The Kopjes are highly rounded exposed metamorphosed granitoid and gneissic rock with rare catenas of very thin soils and occasional colluvial soils with crack infills – and are understudied geographic features (Marchant et al., 2018; Migoń et al., 2017; Prendergast, 2020). Fires are generally uncommon as fuels have low connectivity. rBC contributions to the sites likely are derived from local to regional scale fires as well as potential regional sources of hydrocarbon use (Streets et al., 2017).

### Field methods

For lake sediments, a 21.5 cm surface core was collected in the southeastern sector of Lake Victoria, Mara Region, Tanzania, from 9.5 m water depth and >5 km from the nearest shore near Bulamba Bay, Speke Gulf (SPK7; Figure 1; geographic coordinates 2.19195° S, 33.66975° E; 1135 m asl; WGS84; Courtney Mustaphi et al., 2024, 2025b). The core was collected 13 July 2018 with a gravity corer (6.6 cm internal diameter tubes; Pylonex, Umeå, Sweden; Renberg and Hansson, 2008) deployed from an anchored motorboat (Courtney-Mustaphi et al., 2021; Scaini et al., 2024). The core was vertically extruded by a threaded rod at 0.5 cm intervals into sterile plastic bags (Whirl-pak Nasco, Pleasant Prairie, WI, USA), transported, and refrigerated at 4°C.

For soil material, at Ngong Rock, Moru Kopjes, Serengeti National Park (NGO1; 20 November 2018; 2.71781° S, 34.79449° E; ~1601 m asl), a 37.5 cm deep pit was manually dug into colluvial soil that had accumulated in a crack that was blocked by shrub vegetation (Courtney Mustaphi, 2019). At 2.5 cm deep intervals down the soil pit face, 100–160 g soil was collected into sterile bags from below the thin litter layer to the nonconforming pit base. Samples were dried and refrigerated.

### Laboratory methods

Three paleofire metrics were measured from contiguous subsamples at both sites (SPK7, *n* = 43; NGO, *n* = 15). These proxies cover a particle size range of fire products from several orders of magnitude: rBC, <0.5 μm; microscopic charcoal, >10 μm; and macroscopic charcoal, >100 μm (Figure 1; Conedera et al., 2009; Whitlock and Larsen, 2001). The transport and dispersal of these combustion products varies and thus the potential source areas differ from within teleconnected spatial scales for rBC, to regional scales for microscopic charcoal and extralocal-to-local scales for macroscopic particles (Clark and Patterson, 1997; Peters and Higuera, 2007; Vachula, 2019; Vachula and Rehn, 2023; Figure 1).

### Paleofire proxy measurements

For rBC, each subsample was dried, homogenized with a planetary mill (Fritsch GmbH, Idar-Oberstein, Germany), and analysed using a Single Particle Soot Photometer (SP2; Droplet Measurement Technologies, Longmont, CO, USA; Arienzo et al., 2019; Chellman et al., 2018). In summary, 5 mg of homogenized dry sediments or soil was suspended in 50 mL of 18.2 MΩ water. The samples underwent multiple steps of sonication (Emerson Electric, St. Louis, MO, USA) and shaking to mobilize rBC from the sediment matrix, then allowed to settle for 24 h at 10°C.

Samples were filtered through two sequential 20 and 10 μm stainless steel inline filters to remove sediment particles to prevent the flow lines from clogging. The aqueous sample stream was nebulized by an Apex-Q jet-type nebulizer (ESI, Omaha, NE, USA) coupled to the SP2 instrument. Standard samples of the rBC-like CaboJet 200 were analysed at the beginning of each day and intermittently throughout the day as an external calibration for rBC concentrations. The rBC concentrations for each sample are reported relative to each sample’s sediment dry weight (μg of rBC per g sediment) by correcting raw rBC concentrations using the known dilution of the resuspended dry sediment in water. These concentrations were further corrected for potential iron oxide interferences using the colour ratio.

Palynological microscope slides were prepared to count microscopic (pollen-slide) charcoal (Finsinger and Tinner, 2005; Tinner and Hu, 2003). Wet sediment subsamples of 1 cm^3^ were treated with a sequence of chemical digestions using 10% HCl, 10% KOH, 40% HF and acetolysis (Erdtman, 1960; Faegri et al., 1989) and sieved through a ceramic crucible mesh size of 500 μm (Moore et al., 1991). The digested residues were wet sieved with a soft nylon mesh size of 500 μm and then covered in 85% glycerine and mounted onto a glass microscope slide with a rectangular 22 × 32 mm coverslip. One tablet of *Lycopodium clavatum* spores was added to each sample prior to the chemical treatment to estimate microscopic charcoal concentrations (Stockmar, 1971; batch number 1031 with *n* = 20,848, 1σ = 3457 spores per tablet; University of Lund, manufactured in 2011).

The microscopic charcoal particles of >10 μm, straight and near-angular in shape and with completely opaque black colouration (Tinner and Hu, 2003), were counted under a Leica DM2500 LED illuminated optical microscope at 200× magnification with an ocular lens graticule. A minimum of 200 objects (microcharcoal > 10 μm and *Lycopodium* marker grains) were counted with an additional minimum criterion of 20 *Lycopodium* spores counted per sample (Finsinger and Tinner, 2005).

Macroscopic charcoal analysis was undertaken at contiguous 0.5 cm resolution down core (*n* = 43). Subsamples of 1 cm^3^ wet sediment were wet sieved through a 100 μm mesh. The soil wet sieved relatively readily and the retained fraction (>100 μm) was transferred to a Petri dish with water and manually probed with a metal pick (Hawthorne et al., 2018). The initial analysis of 1 cm^3^ soil samples yielded very low concentrations of charcoal >100 μm and a second analysis was undertaken that used a larger volume (5 cm^3^) (Courtney Mustaphi et al., 2015; Whitlock and Larsen, 2001). Soil samples were dry sieved through a 2000 μm mesh to remove coarser organic and clastic material and visually checked for charcoal. Afterwards, 5 cm^3^ of the sieved matrix was subsampled and wet sieved (>100 μm) and counted using the same method described for lake sediment samples. The concentration values were scaled to charcoal count per 1 cm^3^.

Data were entered into spreadsheets for calculations and Spearman’s rank correlation (*r_s_*) tests done on the original data using R version 4.1.2 base statistical function (R Foundation for Statistical Computing, 2021). Interpretation of the correlations did not rely on *p*-values as these cannot reliably assess significance of linear relationships in non-stationary time series characterized by (varying) long-term trends. To assess whether different paleofire proxies recorded similar trends and if changes exceeded the between-sample variability at different depths, without relying on *p*-values, we pooled the time series into bins. Down profile values were pooled into non-overlapping bins of five samples each to calculate a binned median and standard deviation. This provides an estimate of variations in central tendency and allowed us to visualize between-sample variability down the records.

### Geochronological age determinations

Sediment accumulation rates for the lake sediment core were estimated through gamma spectroscopy of lead-210 and caesium-137 radioisotopes (Appleby et al., 1986) with a Canberra BEGe detector housed at Northeastern University for 11 dried and homogenized sediment subsamples at contiguous 2 cm thick intervals downcore (mean = 1.22, and range = 0.47–1.65 g). Samples were sealed for a minimum of 2 weeks prior to gamma counting (Putyrskaya et al., 2015). Constant rate of supply (CRS) age estimates were produced with 95% confidence intervals from the gamma counts and measured error and corrected with the caesium-137 peak at 6–8 cm (Appleby, 2001). This increase was assumed to represent the AD1963 past nuclear fallout maximum that was caused by the height of aboveground atomic explosions globally (Appleby et al., 1986). Four soil samples were dried (range = 29.7–34.9 g) and analysed for caesium-137 using a broad energy, high purity Germanium (HPGe) detector (Mirion, Rüsselsheim, Germany) at the University of Basel and the detection of caesium-137 may suggest that the sediments are younger than the 1950s–1960s or that there has been vertical mixing of younger material throughout the profile.

## Results

**Figure 2.**
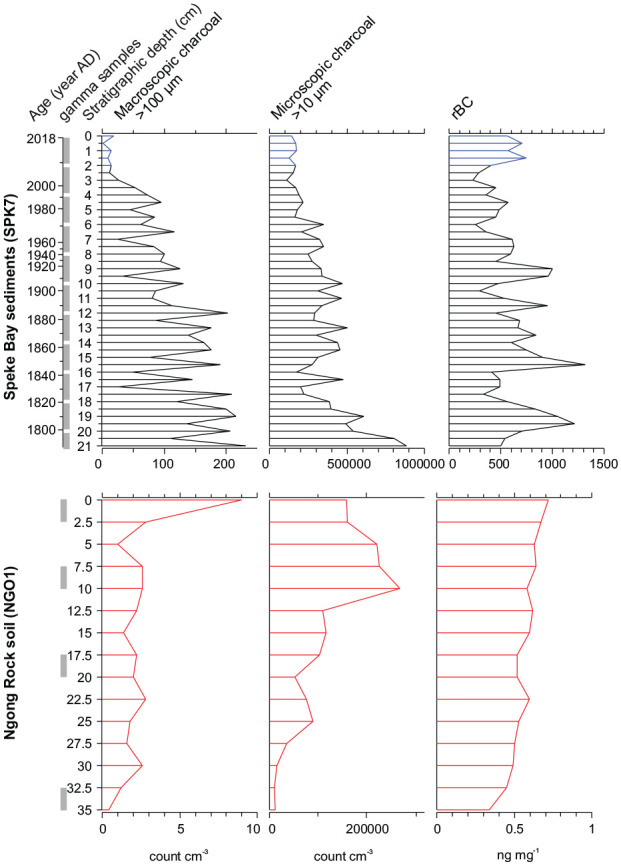
Paleofire proxy results (concentrations) of macroscopic (>100 μm) and microscopic charcoal (>10 μm) and refractory black carbon (rBC) down the lake sediment core and soil pit profile. Arranged from larger particle sizes (left) to smaller (right). The uppermost five samples are highlighted (blue) to refer to Figures 3 and 4. Samples extracted for gamma counts are shown in vertical grey boxes (left). CRS age model estimates (year AD) are shown as secondary *y*-axis for the sediment core SPK7 (see Figure S1). Gamma counts for the four soil samples of NGO1 are shown in Figure S2). *Note*. Please refer to the online version of the article to view this figure in color.

The lake sediment core provided a paleofire record of the past century and half (Figure 2 and S1). For all four soil samples, caesium-137 was detected, which supports that the soil contains a significant amount of material younger than the mid-20th century (Figures S2). The sediment core analyses resulted in a refractory black carbon range of 233–1321 ng mg^−1^ (rBC, mean = 611, σ = 252; Table S1), microscopic charcoal concentrations of 110,558–884,998 pieces cm^−3^ (mean = 326,121, σ = 171,097), and macroscopic charcoal concentrations that ranged from 1 to 232 pieces cm^−3^ (mean = 101, σ = 66). The amount of microscopic and macroscopic charcoal paleofire indicators decreased from the base to the top of the core, while rBC concentrations deviated slightly with an increase at the top, from 2.5 to 0.0 cm (Figure 2, shown in blue). Relatively strong correlations were found between macroscopic and microscopic charcoal concentrations (*r*_s_ = 0.70) and between microscopic charcoal concentrations and rBC concentrations (*r*_s_ = 0.47; Supplemental Figure S3). Macroscopic charcoal and rBC were weakly correlated (*r*_s_ = 0.32).

The soil pit profiles of each proxy generally increased from the base to the surface soil, although in different patterns towards the uppermost layers (Figure 2). The soil stratigraphy had rBC concentrations that ranged from 0.34 to 0.72 ng mg^−1^ (rBC, mean = 0.56, σ = 0.10; Table S1), microscopic charcoal concentrations of 10,840–268,418 pieces cm^−3^ (mean = 110,506, σ = 82,283) that increased in a stepwise pattern to 10 cm and then showed a slight decrease to the surface and macroscopic charcoal concentrations that ranged from <1 to 9 pieces cm^−3^ (mean = 2.4, σ = 2.0) and were relatively stable until an extreme peak at the top. The correlations for the soil are limited by low sample count but the patterns are similar as for the lake sediment record. The correlation between macroscopic and microscopic charcoal concentrations was moderate (*r*_s_ = 0.56) and between microscopic charcoal concentrations and rBC concentrations was strong (*r*_s_ = 0.83; Supplemental Figure S3). The weakest correlation was between macroscopic charcoal and rBC (*r*_s_ = 0.40).

**Figure 3.**
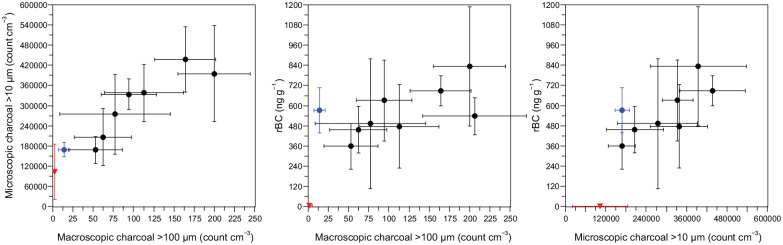
Plot of the stepwise median and 1σ standard deviation values (*n* = 5 sample steps, 2.5 cm intervals) between each combination of the three paleofire metrics: macroscopic charcoal, microscopic charcoal, and refractory Black Carbon (rBC). The colours relate to the stratigraphic profiles shown in Figure 2, with most of the SPK7 sediment core in black and the uppermost samples shown in blue. The median and standard deviation is also shown for the soil site NGO1 (in red, *n* = 15; as in Figure 2). *Note*. Please refer to the online version of the article to view this figure in color.

When down profile data of each paleofire metric were 5-sample pooled, the median values tended to increase with increasing values of any other metric. The standard deviations of very low and very high values tended to not overlap (Figure 3), which reflects the general pattern of decreasing values from profile bases to tops (Figure 2). The soil stratigraphy values were the lowest observed (Figure 3, shown in red) and, for comparisons with rBC, the topmost sediment samples (Figure 3, shown in blue) deviated slightly from the downcore pattern.

**Figure 4.**
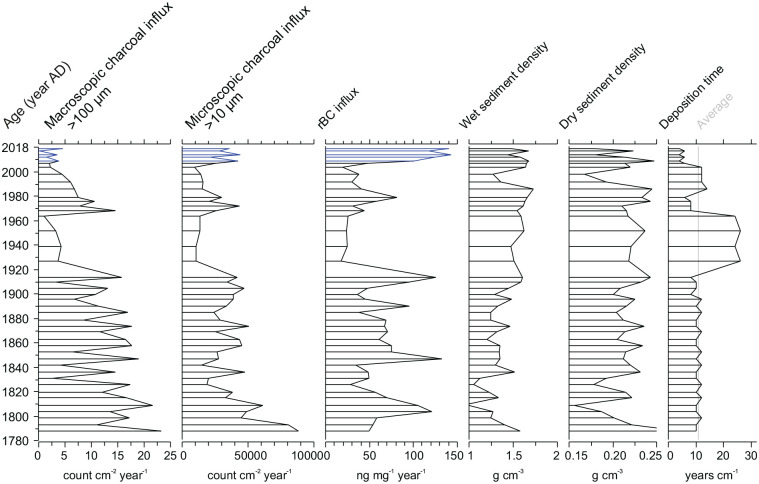
Accumulation rates (influx) of paleofire proxy results from Speke Gulf (SPK7) calculated using the CRS age-depth model.

Sediment accumulation rates averaged 11 years cm^−1^ downcore and varied 5–25 years cm^−1^ since the mid-19th century (Figure 4). Accumulation rates of the paleofire metrics were calculated using the CRS age-depth model for the SPK7 sediment core to explore variations in relation to the variable sediment accumulation rates. Overall downcore patterns are similar to concentration values but are sensitive to larger variations in sediment accumulation rate, notably the decreased rates between AD1910–1960 and 1980–2005 (Figure 4). The topmost samples of rBC show much more prominent peak values in rBC accumulation relative to concentration values (Figure 4). The detection of caesium-137 in all four samples down the soil pit profile suggests contributions of material younger than the mid-20th century and was not used to calculate an age-depth model and paleofire influx values (Figure S2).

## Discussion

The results show that rBC measurements can be applied to sediments of very large lakes and soils in semi-arid tropical ecosystems (Table S1). To better contextualize the relationship between these proxies a correlation and down profile analysis was done to intercompare concentrations of rBC with microscopic and macroscopic charcoal (Figures 2, 3, S1, S2 and S3), which are the two most common paleofire proxies used in depositional environments (Adolf et al., 2018; Bremond et al., 2024; Hawthorne et al., 2018; Marlon et al., 2016). At both of the sites analysed, the results fit with expectations that paleofire proxies reflect a spatial aggregation of fire activity from local to regional scales. Within each site, correlations between each metric were positive and the strongest positive correlations were found between paleofire metrics that are often interpreted to reflect more similar source-area scales, particle size, and transport distances (Figure 1; Brugger et al., 2022). Correlations were strongest between macroscopic and microscopic charcoal representing local-to-regional scales, and between microscopic charcoal and rBC that represent regional, continental and hemispheric scales (Figure S3; Brugger et al., 2023). The weakest correlation was between macroscopic charcoal and rBC, which are often interpreted to represent very different spatial source areas, transport and depositional patterns (Figure 1; Brugger et al., 2024; Conedera et al., 2009).

Published rBC values are useful to inform future studies, but direct intercomparison of rBC values between sites and environmental geoarchives may be misleading without further quantification of the different depositional mechanisms into depositional matrices (soil, sediments, ice) and taphonomic factors. Comparing the Speke Gulf rBC values with other lake sediment records globally, the high rBC concentrations are higher than observed elsewhere, to date, with the exception of tropical lake sites in the Amazon Basin (Table S1; Arienzo et al., 2019). Potentially the large watershed and lake surface area of Speke Gulf are factors that contribute to the high rBC concentrations in the sediments (Bennett and Buck, 2016; Blais and Kalff, 1995; Wetzel, 2001) and syndepositional and other taphonomic processes influencing deposition remain uncertain. The soil rBC concentrations were intermediate, the lowest values correspond to measurements from ice core records and the higher values correspond to the lower measurements from lake sediments (Table S1). Colluvial soils may accumulate relatively quickly (Heckmann, 2014) and the rBC may be more diluted in the NGO1 mineral soil relative to soils that accrue more slowly and over longer timescales, such as some sites from African highlands (Little and Aeolus Lee, 2006; Mahaney et al., 1991; Montade et al., 2018; Ossendorf et al., 2019).

The influx values of the paleofire proxies conserved the overall trend in the concentration values and are dependent on the sediment accumulation rates, notably changepoints in the rates (Figures 2 and 4). Sediment accumulation rates varied and slower sediment accumulation occurred between AD 1910–1960 and 1980–2005 that also had lower paleofire influx values, but the bulk density does not show a major change (Figure 4). The topmost samples of rBC show much more prominent peak values in microcharcoal and rBC accumulation relative to concentration values (Figure 4), potentially relating to increased fires and transport to the core location from the region.

Large basins integrate paleofire indicators that result in a spatially aggregated fire signal and there are few records of microscopic or macroscopic charcoal from very large basins (Daniau et al., 2013; Genet et al., 2021; Temoltzin-Loranca et al., 2023). The Speke Gulf sediments have decreasing paleofire indicator concentrations towards the surface while the soil profile has increasing concentrations. The tested paleofire proxies (rBC, microscopic and macroscopic charcoal) had similar distributions and were positively correlated with coherent within site paleofire proxy patterns (Figure 2 and S3) and a relationship between lower and higher values between variables is evident with pooled samples (Figure 3). The results reflect the spatial and temporal aggregation of the paleofire signals at each site, the large lake basin and a very local soil accumulation. Larger particles of paleofire indicators generally transport a shorter distance than the smaller diameter fire products (Peters and Higuera, 2007; Vachula and Rehn, 2023) and the depositional setting and catchment areas are very different for the lacustrine embayment and colluvial soil (Courtney Mustaphi et al., 2015; Whitlock and Larsen, 2001; Whitlock and Millspaugh, 1996). In contrast, the soil stratigraphy presents a highly local depositional system as a very localized area for the deposition of all paleofire proxies. Macroscopic charcoal at the investigated kopje are likely sourced from the immediate surrounding and microscopic charcoal and rBC from the broadscale savannah, shrublands and forest ecosystems that surrounds the site up to hundreds or thousands of kilometres.

The large continental lake catchment area receives air masses from both Northern and Southern Hemisphere Africa and these new results thus reflect regional scale burning patterns (Kirago et al., 2022). Few lake sediment rBC records have been published and the results show the potential to apply rBC as a large-scale paleofire signal from the sediments of large lakes. An important additional component to the rBC results of Speke Gulf (SPK7) was the slight and sustained increase of values from 2.5 cm to the surface (AD2003–present) that was not observed for micro- and macroscopic charcoal. This difference may be due to the anthropogenic contribution from fossil fuel use and hydrocarbon combustion over the past century in Sub-Saharan Africa (Amos et al., 2013; Cook, 1994; Liu et al., 2021; Wang et al., 2010). Previous work has linked fossil fuel use to observed increases in rBC in environmental archives over the industrial era in both ice (Brugger et al., 2023; McConnell et al., 2007; Osmont et al., 2018; Sigl et al., 2018) and sediment cores (Ruppel et al., 2021).

The results from this study contribute to the literature of multiple paleofire indicator intercomparisons from single site sedimentary and soil records (Courtney Mustaphi and Pisaric, 2014; Rehn et al., 2021; Tan et al., 2015, 2018) and demonstrate the potential of rBC measurements on geoarchives from Africa and the tropics where ice cores are scarce and direct rBC emission and deposition estimates are currently limited (Giglio et al., 2013; Penner et al., 1993). Further paleofire insights and in new study areas will benefit from multiple fire-proxy analyses of co-located samples (Maezumi et al., 2021).

## Conclusions

This study analysed refractory black carbon (rBC) in lake sediments and soil from tropical eastern Africa in relation to microscopic and macroscopic charcoal to test rBC as a paleofire proxy in semi-arid ecosystems. The correlation structure suggests the different paleofire metrics represent different source areas, particle size and transport taphonomies; with relatively strong positive correlations observed between rBC and microscopic charcoal, and between microscopic and macroscopic charcoal. rBC is not as strongly correlated with macroscopic charcoal. These patterns may be explained by different source areas: with macroscopic charcoal integrating local sources, microscopic charcoal integrating extralocal and regional sources and the submicron particles analysed with rBC representing an even larger source area. As expected from theoretical and modelled paleofire transport models, the analyses of an extralocal-regional scale signal in the lake sediments and the local-to-regional scale soil site suggest that paleofire indicators are scale dependent. Further development of rBC records and intercomparison with other paleofire proxies from different depositional settings and sedimentary materials will further refine the paleofire interpretation potential, especially as rBC is applied to additional glacial records and deeper geologic timescales. Differences between rBC and charcoal have potential for disentangling fossil fuel combustion and both natural and anthropogenic biomass combustions during the industrial period and for providing fire reconstructions at different spatial scales. Multidisciplinary and integrated research on emissions from spatial extents and temporal rates, fire types, modern accumulation rates and retrospective studies that use paleoenvironmental archives will further develop the fire-proxy relationships.

## Supplemental Material

sj-docx-1-hol-10.1177_09596836251340882 – Supplemental material for Multiple paleofire proxy metrics from tropical lake sediment and soil in the Greater Serengeti Ecosystem Supplemental material, sj-docx-1-hol-10.1177_09596836251340882 for Multiple paleofire proxy metrics from tropical lake sediment and soil in the Greater Serengeti Ecosystem by Colin J. Courtney Mustaphi, Sandra O. Camara-Brugger, Nathan J. Chellman, Samuel Muñoz, Rebecca W. Kariuki, Maja Damber, Anna Shoemaker, Anneli Ekblom, Linus Munishi, Paul Lane, Rob Marchant and Oliver Heiri in The Holocene
