# Supplementary material for: Multiple paleofire proxy metrics from tropical lake sediment and soil in the Greater Serengeti Ecosystem
Source: Holocene. 2025 Jun 26;35(9):926–36. doi: 10.1177/09596836251340882 (PMC12342397; doi:10.1177/09596836251340882)

**Supplementary Information**

**Supplementary Table S1.** A list of refractory black carbon (rBC) concentration ranges from previously published study sites globally. The accumulation of different environmental matrices (soil, sediments, ices) precludes direct comparisons amongst all identified sites and these concentration measurements are presented for metrologic comparison to inform future studies.

| **Geographic area** | **Archive type** | **rBC concentration range (ng mg^-1^)** | **Reference** |
| --- | --- | --- | --- |
| eastern Africa | soil pit | 0.34–0.72 | this study, Ngong Rock (NGO1) |
| eastern Africa | lake sediments | 233.19–1321.32 | this study, Lake Victoria (SPK7) |
| Amazon Basin | lake sediments | 300–8900 | Arienzo et al., 2019 |
| New Zealand | lake sediments | 0.85–390 | Brugger et al., 2024 |
| Patagonia | lake sediments | 4–214 | Camara-Brugger et al., 2025 |
| Wyoming, USA | lake sediments | 100–300 | Chellman et al., 2018 |
| Siberia | lake sediments | 0–300 | Chellman et al., 2018 |
| Bolivian Andes | High-Alpine glacier | 0.0002–0.0130 | Osmont et al., 2019 |
| Antarctica | Ice Sheet | 0.000025–0.001000 | McConnell et al., 2021 |
| Greenland | Ice Sheet | 0.0002–0.0040 | Brugger et al., 2023 |

**Supplemental information on gamma counting and CRS age-depth model of sediments**

Sedimentological and paleofire indicator measurements for the SPK7 sediment core collected from Speke Gulf, Lake Victoria, Tanzania. Sediment accumulation rates were estimated through gamma spectroscopy of lead-210 and caesium-137 radioisotopes (Appleby et al., 1986) with a Canberra BEGe detector housed at Northeastern University for 11 dried and homogenised sediment subsamples at contiguous 2 cm thick intervals downcore (mean=1.22 g, range=0.47–1.65 g). Samples were sealed for a minimum of two weeks prior to gamma counting (Putyrskayaet al., 2015). Constant rate of supply (CRS) age estimates were produced with 95% confidence intervals from the gamma counts and measured error and corrected with the caesium-137 peak at 6–8 cm (Appleby, 2001) and assumed to represent the 1963 AD past nuclear fallout maximum (de Lima Ferreira et al., 2016). The results suggest the sediment accumulation for the core is <200 years (Supplemental Figure S1 and see Courtney Mustaphi et al., 2025b).

Soil samples were collected from a pit on Ngong Rock, Moru Kopjes, Serengeti National Park, Tanzania (Supplemental Figure S2). Four samples of 2.5 cm thickness each were dried and sealed for three weeks prior to gamma counting (Putyrskayaet al., 2015). Caesium-137 counts were measured at Geoecology, University Of Basel, Switzerland, and used to assess the potential age of the soils. The presence of Caesium-137 throughout the samples suggests the soil is very young, since the onset of atmospheric bomb detonations, or the soil has been subject to vertical mixing (bioturbation and physical).

**Supplementary References**

Appleby PG (2001) Chronostratigraphic techniques in recent sediments. Tracking environmental change using lake sediments. Volume 1. Basin analysis, coring, and chronological techniques. Dordrecht NL, Springer. pp.171–203.

Appleby PG, Nolan PJ, Gifford, et al. (1986) 210Pb dating by low background gamma counting. Hydrobiologia 143: 21–27.

Arienzo MM, Maezumi SY, Chellman NJ et al. (2019) Pre-Columbian fire management linked to refractory black carbon emissions in the Amazon. Fire 2(2): 31.

Brugger SO, McWethy DB, Chellman NJ et al. (2024) Holocene black carbon in New Zealand lake sediment records. Quaternary Science Reviews: 108491.

Brugger SO, Chellman NJ, McConnell C et al. (2023). High-latitude fire activity of recent decades derived from microscopic charcoal and black carbon in Greenland ice cores. The Holocene 33(2): 238–244.

Camara-Brugger SO, McWethy DB, Chellman NJ et al. (2025) Patagonia's Late Holocene lake sediments reveal no major black carbon sources for Antarctica. Anthropocene 49: 100458.

Chellman NJ, McConnell JR, Heyvaert A et al. (2018). Incandescence‐based single‐particle method for black carbon quantification in lake sediment cores. Limnology and Oceanography: Methods 16(11): 711–721.

Courtney Mustaphi CJ, Brugger SO, Ekblom A et al. (2025b) Multiporate Poaceae pollen grains observed in the recent fossil record from the Greater Serengeti Ecosystem and Lake Victoria region. Review of Palaeobotany and Palynology 333: 105240.

de Lima Ferreira PA, Figueira RCL, Siegle E et al. (2016) Using a cesium-137 (137Cs) sedimentary fallout record in the South Atlantic Ocean as a supporting tool for defining the Anthropocene. Anthropocene 14: 34–45.

McConnell JR, Chellman NJ, Mulvaney R et al. (2021) Hemispheric black carbon increase after the 13th-century Māori arrival in New Zealand. Nature 598(7879): 82–85.

Osmont D, Wendl IA, Schmidely L et al. (2018). An 800-year high-resolution black carbon ice core record from Lomonosovfonna, Svalbard. Atmospheric Chemistry and Physics 18(17): 12777–12795.

Putyrskaya V, Klemt E, Röllin S, Astner M, Sahli H (2015) Dating of sediments from four Swiss prealpine lakes with 210Pb determined by gamma-spectrometry: progress and problems. Journal of Environmental Radioactivity 145: 78–94.

**Supplemental Figure S1.** Gamma counts with 95% CI uncertainty estimates of 2-cm intervals of dried sediments and high resolution (contiguous 0.5-cm intervals) co-located paleofire proxies measured in the Speke Gulf (SPK7) sediment core: macroscopic charcoal, microscopic charcoal, refractory black carbon (rBC). The sediments have a wet sediment density to dry sediment weight ratio between 6–8 and were aggregated to 2-cm thicknesses for gamma counting (left side, plotted by sample top depth) (Courtney Mustaphi et al., 2025b). The ^210^Pb background was reached below 14–16 cm. Peak ^137^Cs was detected at 6–8 cm depth and represented the peak atomic bomb testing around the year 1963. The downcore 210Pb profile suggests continuous sediment accumulation within 95% CI. The average sediment accumulation rate for core is 11 yr cm^-1^.


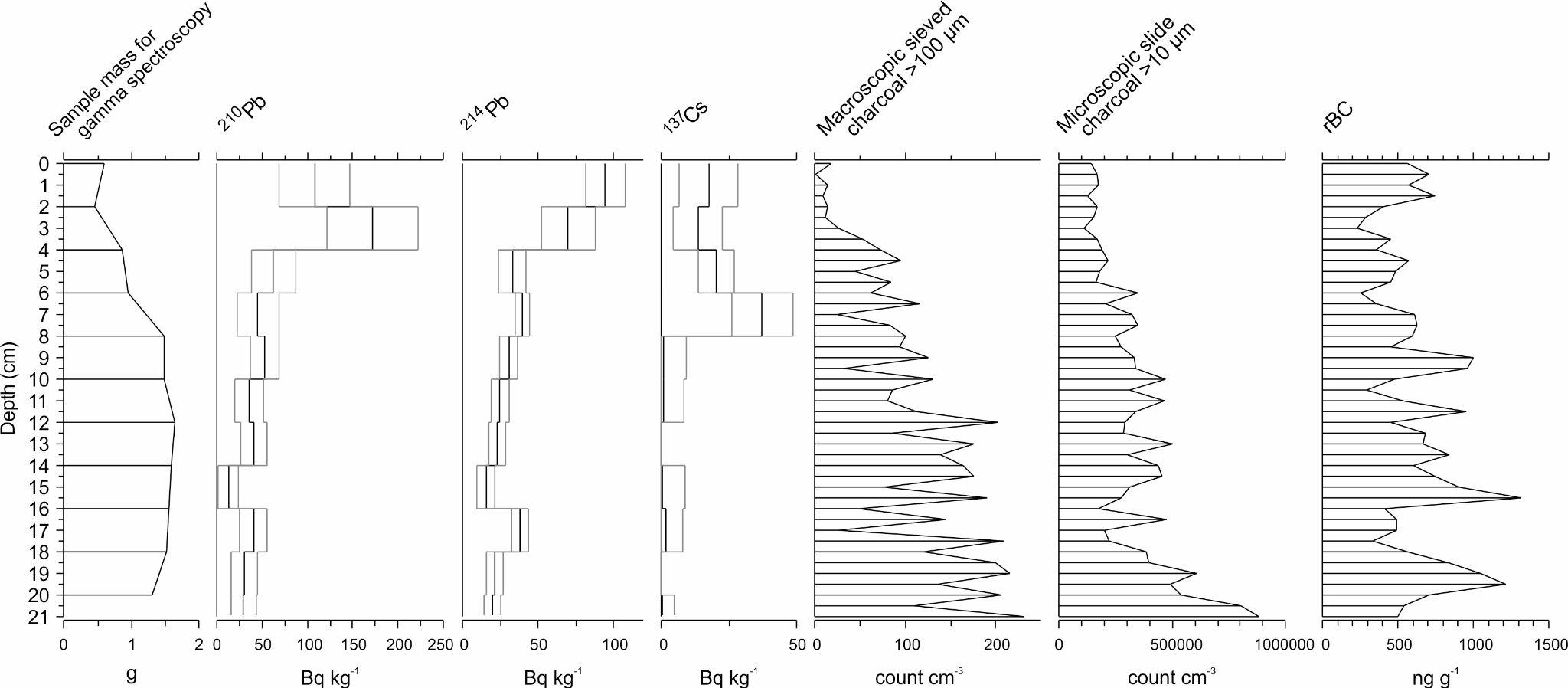


**Supplemental Figure S2.** Gamma counts of ^137^Cs (black bars) with 95% CI uncertainty estimates (grey bars) of 4 samples (2.5-cm thickness) and paleofire proxies measured in the Ngong Rock (NGO1) soil pit: macroscopic charcoal, microscopic charcoal, refractory black carbon (rBC).


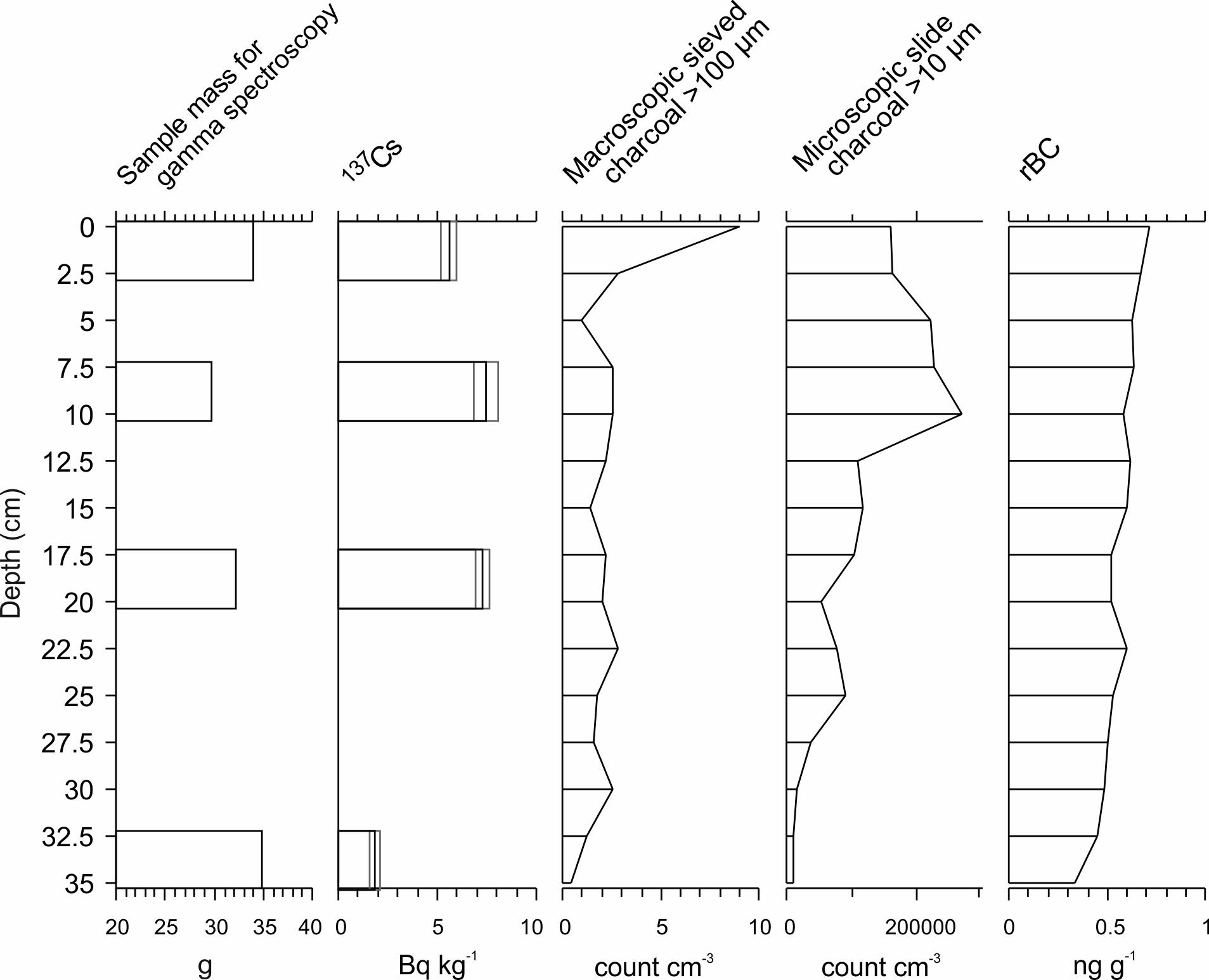


**Supplemental Figure S3.** Scatterplots and Spearman’s rank correlation (rs) for the n=43 co-located paleofire proxies measured in the Speke Gulf (SPK7) sediment core and Ngong Rock soil pit (NGO1). Note that p-values are not presented here because of problems related to geologic time aggregation effects that lead to non-independence between samples in the stratigraphy.


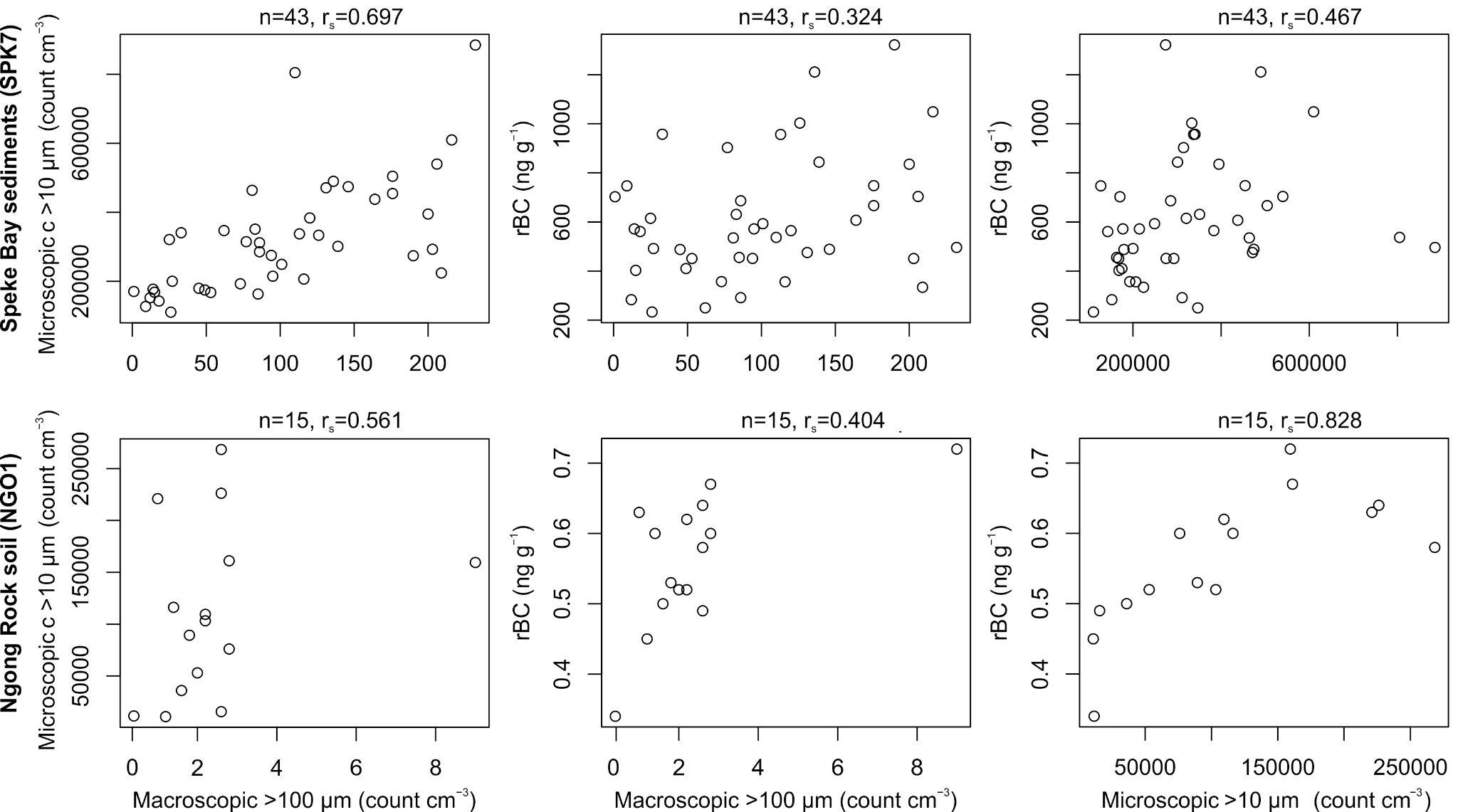

Supplement: sj-docx-1-hol-10.1177_09596836251340882 – Supplemental material for Multiple paleofire proxy metrics from tropical lake sediment and soil in the Greater Serengeti Ecosystem [file sj-docx-1-hol-10.1177_09596836251340882.docx]
